# Biosimilar Insulins and Their Impact on Prices and Utilization of Insulins in Bulgaria

**DOI:** 10.3390/healthcare9060697

**Published:** 2021-06-09

**Authors:** Konstantin Tachkov, Zornitsa Mitkova, Petya Milushewa, Guenka Petrova

**Affiliations:** Faculty of Pharmacy, Medical University of Sofia, 1431 Sofia, Bulgaria; ktashkov@pharmfac.mu-sofia.bg (K.T.); sppmitkova@mail.bg (Z.M.); petya.milushewa@gmail.com (P.M.)

**Keywords:** biological, biosimilar, insulin, competition, prices, utilization

## Abstract

The aim was to explore the availability of biosimilar insulins on the national market in Bulgaria and their impact on prices and utilization. This was a retrospective, quantitative, longitudinal study during the period 2014–2020. Authorized-for-sale, biosimilar insulins at the European level were compared with those on the national market. Prices and utilization were compared in value, number of defined daily dose (DDD), and DDD/1000 inh/day. Almost all types of insulins possessed biosimilars, and even more than one on the European market, but only two were found to be available and reimbursed on the national market. The total number of reimbursed INNs was 11, and for seven of them, changes in reference price per DDD were found. The highest price decrease was observed for insulin (price per DDD decline from 2.77 to 2.22 Bulgarian Leva (BGN)). The total expenditure for insulin increased from 68 to approximately 72.8 mil BGN (34 to approximately 37 mil Euro). The utilization in DDD/1000/inh/day decreased from 16.12 to 15.31. Only two biosimilar insulins were found to be available on the national market, with a slow decrease in prices and stable utilization. Other regulatory and financial measures are probably necessary to foster the insulins’ competition at the biosimilar level.

## 1. Introduction

The successful results of introducing generic medicines worldwide have led to a tremendous decrease in prices and increase in utilization of low priced, with comparative quality, generics [1]. It creates optimistic expectations about the impact of biosimilar medicines on the international markets [2].

Many countries have introduced a variety of policy measures to stimulate the market entrance of biosimilars and to foster the competition at that level [3,4]. Because biosimilars should be distinguished from synthetic generics, due to their higher development costs, differences in the manufacturing process, and different development requirements, one can expect to have different competition at the biotechnology level [5,6]. Some authors have reported that, despite the significant diversity in biosimilar and generic markets, a similar tendency is observed regarding the factors influencing generic and biosimilar uptake [7,8].

The influence of the positive factors stimulating the biosimilar uptake on the market makes it rapidly evolving and attractive for the application of enhancing policies. Nevertheless, a wide difference in biosimilar uptake has been observed across Europe and among the therapeutic biosimilar classes [9].

Insulins are not an exception from the overall tendency of developing and authorizing for sale of biosimilars, especially because most of the insulin until now has been manufactured and distributed by a limited number of multinational companies [10]. Chronic and long-lasting diabetes type 1, for which insulins are a major and only source for therapy, makes physicians more conservative in initiating any change in therapy. Prescription habits of physicians are not mainly driven by economic considerations in cases of type 1 diabetes therapy. Therefore, we can expect that the market uptake of biosimilar insulin might not follow the general tendencies, which stimulated our interest in this study.

Bulgaria is not an exception from the overall world tendency of the diabetic population increase. Nearly 500,000 diabetics are registered and, out of them, between 15% to 18% are insulin users [11]. The cost of diabetes therapy is constantly rising and presents a heavier burden for the health insurance institution [12]. Therefore, studying the insulin utilization and prices might provide in-depth information for future policy measures.

Our main aim was to explore the availability of biosimilar insulins on the national market in Bulgaria and their impact on prices and utilization.

## 2. Materials and Methods

### 2.1. Design of the Study

This was a retrospective, quantitative, longitudinal study of the utilization and price changes of available insulins on the national market during the period of 2014–2020. Three national and one international database were searched.

### 2.2. Data Sources

From the database of European Medicines Agency (EMA), information was extracted about biosimilar insulins and date of their authorization on the European market [13].

The database of National Council of Prices and Reimbursement (NCPR) was searched for information about the changes in prices of reimbursed insulins during the period of interest [14]. The reference price per defined daily dose (DDD), that is the lowest price per DDD within the same INN, was considered.

Information for the utilization of insulin in number of pens and expenditure was extracted from the National Health Insurance Fund (NHIF) database [15].

The number of inhabitants was taken from the National Statistical Institute database for every year of observation [16].

The study used officially available datasets, and no ethical approval was required.

### 2.3. Utilization and Price Analysis

Changes in prices were analyzed in reference price per DDD.

The utilization in number of pens was converted to DDD per International Nonproprietary Name (INN) and analyzed as an absolute value from the beginning until the end of the observed period.

The utilization in DDD/1000 inh/day was calculated following the formula: DDD/1000 inh/day = ((Utilization in n DDD/365/n of inhabitants) ∗ 1000) [17].

## 3. Results

### 3.1. Biosimilars Availability

At the beginning of April 2021, the EMA had authorized for sale seven biosimilar insulins, and one had been refused (Solumarv) [13]. Almost all types of insulin possess authorized biosimilar alternatives, and even more than one product, indicating the increasing competition on the European market. In contrast, on the national market, only two biosimilar insulins were found to be included in the positive drug list and reimbursed with the delay of five years for the first authorized product (Abasaglar). The second one (Lyumjev) had a delay of only seven months after its European authorization. Nevertheless, five biosimilar insulins were unavailable on the national reimbursement market, and there was no strong competition on that level (Table 1).

### 3.2. Changes in Prices

The total number of reimbursed INNs was 11, and for seven of them, changes in reference price per DDD were found (Table 2).

The significant changes of reference price per DDD were not found during 2014–2020 in Bulgaria. The highest price decrease was observed for insulin glargine, where the reference price per DDD declined from 2.77 to 2.22 BGN during 2014–2020, as well as Insulin degludec, with a reference price change from 5.4 to 3.35 BGN in 2016 and 2020, respectively. Those price changes were mostly due to the administrative price revision because of decreasing prices in the reference countries. Only for insulin glargine we can consider that the price decrease might have been due to biosimilar entrance.

### 3.3. Changes in Utilization

The total expenditure for insulin increased from 68 to approximately 72.8 mil BGN (34 to approximately 37 mil Euro) during 2014–2020, even though two insulins dropped down from reimbursement (Table 3). In 2019, the expenditure for insulin glargine exhibited a small decrease. The second biosimilar became available at the end of 2020, and it could not have influenced the utilization for the last two months of the year.

**Figure 1:**
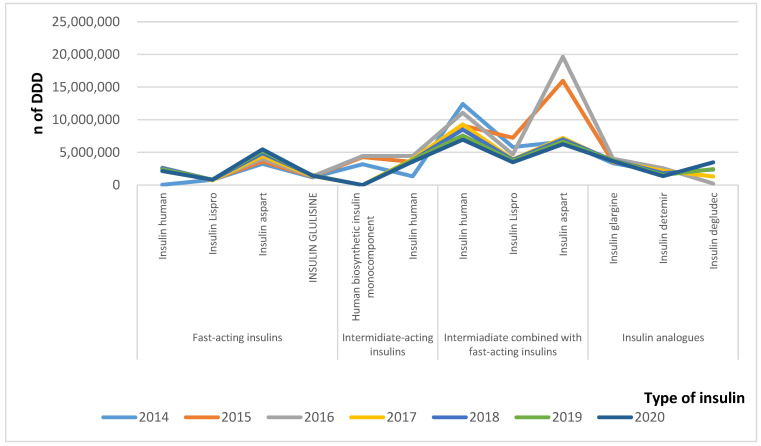
Utilization of insulins in DDD.

The changes in utilization, measured as the number of defined daily doses, are shown in Figure 1. It is evident that the utilization of intermediate- combined with fast-acting insulins prevailed and that the general tendency was the decrease in the number of utilized DDDs, from 42 to 39 mil, in contrast with the rising expenditures.

A possible reason could have been the changes in prices or relative share of utilized insulins. The second assumption is evident by the fact that the number of utilized DDDs of highly prices insulin analogues increased from 5.3 to 8.5 mil DDDs. Fast-acting insulins also increased their utilization in DDDs from 8.6 to 9.8 mil DDDs, and all other subgroups decreased their utilization for the observed period (Figure 1).

**Figure 2:**
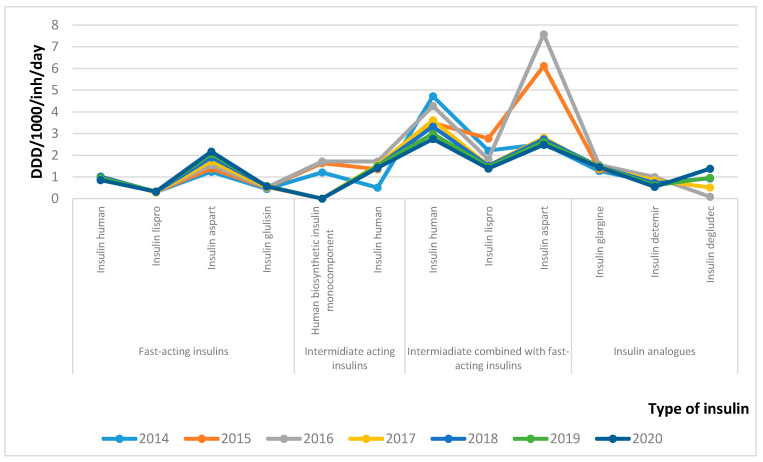
Utilization of insulin in DDD/1000 inh/day.

Following the tendency of utilization in numbers of DDD, the utilization in DDD/1000/inh/day also exhibited a decrease for the period from 16.12 to 15.31 (Figure 2). The utilization of insulin analogues and fast-acting insulins increased, but the other type of insulins decreased, thus forming a negative tendency in utilization. Such a tendency was probably formed by the transfer of most of the patients on an intensified therapeutic regime.

## 4. Discussion

The introduction of biosimilar insulin could support the access to affordable therapy [18]. The first available biosimilar insulin entered the EU during 2015, and in the same year, it was available in most of the EU countries. A total of 18 out of 24 countries funded Abasaglar biosimilar insulin glargine in 2017. Internal reference pricing or prescriber incentives about biosimilars were introduced in all EU countries within this period [3]. Those measures could have affected the supply-side policies for promoting access to biosimilars. Recommendations for investments were made, as well as communication on biosimilars and education of the stakeholders. What has been highlighted is the need for physician information on the entry and use of biosimilars. It would increase the trust in their effectiveness and interchangeability. The initial price decrease of biosimilars as part of the originator could also be used as incentive to prescribe them. Risk sharing agreements has been recommended as an incentive to prescribe, dispense of, and use biosimilars. Binding quota have also been used to support a sustainable biosimilar market [3].

In the middle of 2020, the market share of biosimilar versus the total insulin market was 4% average for the EU market [19]. During 2014–2020, we found only two trademarked biosimilar insulins approved and available on the Bulgarian market. We could consider that the Bulgarian patients do not have sufficient access to biosimilar insulins and that there is still no biosimilar competition available in the segment of insulins.

Biosimilar prices are lower than that of the originator product, and strong competition between insulin manufacturers brings down the prices more efficiently [20]. Comparing the price per patient revealed that increasing competition in insulin manufacturing led to large price reductions, potentially enabling the scale-up of access to treatment [21]. Estimated prices of biosimilar insulins were found to be lower than the prices of insulin analogues.

The slow entrance of biosimilar insulins led to almost unchanged number of INNs in Bulgaria and relatively stable medicine prices. Limited competition on the Bulgarian market did not notably impact overall insulin utilization and NHIF payment during the study period. On one hand, the higher variation in utilization was found for intermediate-acting insulin combined with fast-acting, especially insulin aspart and human insulin. The highest change in reference price per DDD was found for insulin analogues. It confirmed that the price change was mainly affected by external reference pricing, not by market competition due to limited number of biosimilar insulins in Bulgaria. We found that, for the earlier biosimilar products, the prices decreased, which partly supports our main conclusion. We also considered that longer observation is needed, but the fact that the prices have remained stable for six years for the rest of the insulins indicates that the available regulatory measures are not sufficient. 

A study in Albania revealed the highest rate of utilization in DDD/1000 inh/day of fast-acting, followed by intermediate-acting combined with fast-acting insulins during 2004–2014 [22].

Worldwide, biosimilar insulin has not been widely used as an originator. A study among Asian countries (India, China, Vietnam, and the Philippines) revealed that only 7.4% of patients were treated with biosimilar insulin in 2018 [23]. Implementation of incentive policies and the date of first biosimilar market entry correlated to biosimilar uptake, while the impact of price discounts was not established [24].

The introduction in Bulgaria of price measures as internal reference pricing within the INN, the price of biosimilars not higher than 80% of the originator price, financial arrangements, and discount could favor biosimilars introduction, but a lack of the other measures as substitution and prescriber incentives could delay biosimilar competition.

Overall, competition between biologics and biosimilars provides not only access to affordable therapy for patients, but it may also stimulate innovation in the development of new next-generation biological products [25].

To the best of our knowledge, this study has been the first one in Bulgaria longitudinally exploring the insulin utilization, price changes, and biosimilars availability, which is its main strength. Its internal validation is supported by the stable tendencies in the utilization patterns, and its external validation is supported by the other studies reporting similar results.

The limitation of our study is the fact that it included only outpatient utilization financed by the public fund in Bulgaria, but there was no available data about insulin used in hospital treatment or out of pocket payment. In general, when hospitalized, diabetic patients bring their own insulin. Only in the case of initiation of therapy may the hospitals supply some quantities.

## 5. Conclusions

Biosimilar insulins have been authorized for sale on the European market for the last seven years, but only two of them were found to be available on the national market. We found small decreases in prices, mostly due to other regulatory measures, and stable utilization. Other regulatory and financial measures are probably necessary to foster the insulins competition at the biosimilar level.

## Figures and Tables

**Table 1:** Authorized-for-sale by EMA biosimilar insulins (April 2021).

Type of Insulin	Trade Name of Biosimilar	Trade Name of Originator	Date of Approval by EMA	Available on the National Market
insulin glargine	Abasaglar	Lantus	9 September 2014	2 November 2019
insulin glargine	Lusdana	Lantus	1 April 2017, withdrawn 29 October 2018	n.a.
insulin glargine	Semglee	Lantus	27 March 2018	n.a.
insulin lispro	Admelog	Humalog	19 May 2017	n.a.
insulin lispro	Lyumjev	Humalog	24 March 2020	2 October 2020
insulin aspart	Sar-Asp	NovoRapid	June 2020	n.a.
Insulin aspart	Kixelle	NovoRapid	February 2021	n.a.

**Table 2:** Reference price per DDD of INNs of insulins approved in Bulgaria.

Type of Insulin	INN	2014	2015	2016	2017	2018	2019	2020
Fast-acting insulin	Insulin human	1.18667	1.10853	1.10853	1.03173	1.03173	1.03173	1.03173
	Insulin lispro	1.89787	1.89787	1.79987	1.89787	1.89787	1.89787	1.89787
	Insulin aspart	1.84960	1.84960	1.84960	1.84960	1.84960	1.84960	1.84960
	Insulin glulisine	1.48453	1.48187	1.45547	1.43067	1.43067	1.40667	1.40667
Intermediate-acting	Insulin human	1.18667	1.10853	1.10853	1.03173	1.03173	1.03173	1.03173
Intermediate-combined with fast-acting	Insulin human	1.18667	1.10853	1.10853	1.03173	1.03173	1.03173	1.03173
	Insulin lispro	2.90907	2.90907	2.90907	2.90907	2.90907	2.90907	2.90907
	Insulin aspart	1.85507	1.85507	1.85507	1.85507	1.85507	1.85507	1.85507
Insulin analogues	Insulin glargine	2.77867	2.70400	2.57107	2.24693	2.24693	2.23840	2.22707
	Insulin detemir	3.30000	3.26400	3.26400	3.26400	3.26400	3.26187	3.24173
	Insulin degludec	0	0	5.40640	4.58907	3.93280	3.71253	3.35573

**Table 3:** Expenditures paid by the NHIF for insulins during 2014–2020 (BGN).

Type of Insulin	INN	2014	2015	2016	2017	2018	2019	2020 (6 Months)
Fast-acting insulins	Insulin human	2,712,974	2,746,003	2,743,330	2,634,612	2,659,802	2,485,864	1,110,865
	Insulin lispro	1,439,959	1,505,685	1,551,176	1,385,607	1,513,013	1,610,551	772,072
	Insulin aspart	6,012,484	6,661,873	7,431,574	8,198,453	8,994,654	9,667,668	5,059,650
	Insulin glulisine	1,721,718	1,887,274	1,903,362	1,996,637	2,113,265	2,085,594	1,003,911
Intermiddiate-acting	Human biosynthetic insulin mono-component	3,525,661	4,729,230	4,573,895	0	0	0	0
	Insulin human	1,474,536	3,929,373	4,573,895	4,086,315	3,939,394	3,949,838	0
Intermiadiate- combined with fast-acting	Insulin human	13,756,838	10,111,514	11,419,348	9,591,356	8,756,552	7,829,981	1,846,691
	Insulin lispro	10,471,092	13,028,771	13,666,035	11,410,897	11,212,032	10,772,541	
	Insulin aspart	12,292,976	29,521,937	36,423,518	13,373,571	12,939,875	12,280,771	3,584,212
Insulin analogues	Insulin glargine	8,598,188	9,046,884	9,050,478	8,130,955	8,437,987	8,392,459	5,084,172
	Insulin detemir	6,432,483	7,715,453	8,420,110	7,134,602	5,953,854	5,052,526	5,811,209
	Insulin degludec			1,066,595	5,218,684	8,823,325	8,177,562	0
Total		68,438,910	90,883,997	102,823,316	73,161,688	75,343,753	72,305,354	4,070,907

## Data Availability

The study used data from officially available sources, and their web pages have been provided in the reference list.
